# Lung ultrasound can predict response to the prone position in awake non-intubated patients with COVID‑19 associated acute respiratory distress syndrome

**DOI:** 10.1186/s13054-021-03472-1

**Published:** 2021-01-25

**Authors:** Sergey N. Avdeev, Galina V. Nekludova, Natalia V. Trushenko, Natalia A. Tsareva, Andrey I. Yaroshetskiy, Djuro Kosanovic

**Affiliations:** Department of Pulmonology, I.M. Sechenov First Moscow State Medical University (Sechenov University), Moscow, Russia

To the Editor,

Prone positioning (PP) is a well-known therapeutic strategy used in acute respiratory distress syndrome (ARDS). Several studies demonstrated positive effects of PP on oxygenation parameters in awake non-intubated patients with COVID-19-associated ARDS [1–3]. However, PP is not effective in every case. The pilot study by Elharrar et al. demonstrated a significant improvement of oxygenation parameters during PP in only 25% of the patients [3]. The results of previous studies highlighted heterogeneity of COVID-19-associated ARDS, which demands further studies of the predictors of PP effectiveness and indications for its use in COVID-19 patients. The main objective of our study was to evaluate whether the changes of lung aeration assessed by lung ultrasound (LUS) can predict the oxygenation response during PP.

This prospective cohort study was conducted in COVID-19 care units of two university-affiliated hospitals (Sechenov University) between April 8 and May 10, 2020. The study included spontaneously breathing patients with confirmed or suspected diagnosis of COVID-19, and bilateral changes detected by high-resolution computed tomography and PaO_2_/FiO_2_ < 300 mmHg.

The study included 22 COVID-19 patients. Median age was 48.5 (39.8–62.8) years, 16 were male, and the median body mass index was 28.7 (27.3–31.6)kg/m^2^. The main co-morbidities were arterial hypertension (31.8%) and diabetes mellitus (18.2%). Sixteen patients (72.7%) received CPAP and 6 patients (27.3%) received oxygen therapy.

Sixteen of 22 patients (72.7%) responded to PP treatment with significant increase in PaO_2_/FiO_2_. At the same time, fewer patients had clinically significant improvement in dyspnea score—3 patients (13.6%) at 15 min in PP and 12 patients (54.5%) at 3 h in PP (Table 1). RR also significantly improved in responders.

**Table 1:** Comparison of changes over time in respiratory variables in responders and non-responders

Parameters	Non-responders	Responders	*p* value
LUS (total aeration) score, baseline	18.5 (16.0–20.3)	17.5 (17.0–20.8)	0.97
LUS (total aeration) score, PP at 3 h	16.0 (14.5–18.8)	13.5 (12.3–14.0)	0.03
LUS (posterior segments) score, baseline	6.0 (4.3–7.3)	8.5 (7.3–9.8)	0.006
LUS (posterior segments) score, PP at 3 h	5.5 (4.0–6.0)	4.0 (4.0–5.0)	0.20
LUS (anterior segments) score, baseline	6.0 (5.3–7.5)	5.0 (4.0–5.0)	0.05
LUS (anterior segments) score, PP at 3 h	6.5 (4.3–7.3)	5.0 (4.0–6.0)	0.11
LUS (lateral segments) score, baseline	6.0 (4.8–7.5)	5.5 (4.0–6.0)	0.37
LUS (lateral segments) score, PP at 3 h	5.0 (4.5–7.3)	4.0 (3.0–5.0)	0.07
PaO_2_/FiO_2_ at baseline	138 (113–177)	136 (118–172)	0.53
PaO_2_/FiO_2_ PP at 3 h	148 (128–182)	181 (174–210)	0.03
PaCO_2_ at baseline	36 (34–41)	37 (34–40)	0.94
PaCO_2_ PP at 3 h	36 (34–40)	37 (35–38)	0.92
SpO_2_/FiO_2_ at baseline	181 (176–228)	180 (177–211)	0.86
SpO_2_/FiO_2_ PP at 15 min	183 (178–230)	190 (188–222)	0.07
SpO_2_/FiO_2_ PP at 3 h	185 (178–224)	194 (193–233)	0.07
SpO_2_/FiO_2_ supine at 15 min	182 (179–226)	188 (184–227)	0.08
SpO_2_/FiO_2_ supine at 1 h	179 (176–226)	184 (182–215)	0.13
RR at baseline	23 (22–26)	24 (20–26)	0.91
RR PP at 15 min	22 (19–26)	21 (20–24)	0.65
RR PP at 3 h	21 (18–27)	19 (16–21)	0.08
RR supine at 15 min	23 (22–26)	20 (18–23)	0.02
RR supine at 1 h	24 (22–26)	23 (18–25)	0.29
HR at baseline	79 (72–93)	81 (79–94)	0.24
HR PP at 15 min	91 (73–100)	88 (76–98)	0.12
HR PP at 3 h	85 (79–97)	74 (70–91)	0.20
HR supine at 15 min	81 (76–88)	89 (80–97)	0.88
HR supine at 1 h	86 (75–104)	79 (74–88)	0.37
Dyspnea Borg at baseline	5 (3–6)	5 (3–6)	0.89
Dyspnea Borg PP at 15 min	4 (2–6)	4 (2–6)	0.95
Dyspnea Borg PP at 3 h	5 (3–7)	3 (2–4)	0.26
Dyspnea Borg supine at 15 min	4 (3–6)	3 (2–6)	0.43
Dyspnea Borg supine at 1 h	5 (4–6)	4 (2–5)	0.12

The study protocol included the measurement of SpO_2_, respiratory rate (RR), heart rate (HR) and dyspnea assessment using Borg-Dyspnea-Scale (at baseline, after 15 min in PP, after 3 h in PP, 15 min and 1 h after turning in supine position). Arterial blood gas analysis was measured twice: at baseline and after 3 h in PP. The increase of PaO_2_/FiO_2_ by 20 mmHg in 3 h after turning a patient into the prone position was used as the criterion of the response to PP. All parameters of respiratory support and FiO_2_ were the same during supine and prone positions. Before PP and after 3 h in PP semi-quantitative assessment of the lung tissue was performed by LUS. The study protocol included 14 areas for scanning (two anterior, two lateral and three posterior regions of each hemithorax) [4]

Data are expressed as median (inter-quartile range). PaO_2_/FiO_2_ (mmHg): arterial oxygen tension to inspired oxygen fraction ratio; PaCO_2_ (mmHg): arterial carbon dioxide tension; SpO_2_/FiO_2_: arterial oxygen saturation to inspired oxygen fraction ratio; RR (min^−1^): respiratory rate, HR (min^−1^): heart rate; LUS: lung ultrasound; PP: prone position

Responders and non-responders demonstrated significant differences in disease duration (8.5 (5.0–10.8) vs. 13.0 (10.0–17.0) days of disease, *p* = 0.02), no other differences in baseline clinical and laboratory parameters were observed. Three patients (all from non-responder group) were transferred to intensive care unit and then intubated, two of them died.

The patients who responded to PP had more pronounced disturbances of aeration in posterior regions (8.5 (7.3–9.8) vs. 6.0 (4.3–7.3); *p* = 0.006) as reflected by greater LUS. The decrease of the total LUS score and LUS score of posterior regions was significantly greater in responders (5.0 (4.0–7.0) vs. 1.5 (1.0–3.0); *p* < 0.005 and 4.0 (3.5–5.0) vs. 1.0 (0.0–1.0); *p* < 0.001, respectively). The area under the receiver operating characteristic curve of posterior LUS score for the oxygenation response during PP was 0.87 (95% CI 0.64–1.0; *p* < 0.01). Changes of aeration score over time in posterior segments by LUS data correlated with PaO_2_/FiO_2_ changes (*r* = 0.53, *p* = 0.01), i.e. aeration improvement in posterior lung segments was associated with improved oxygenation status (Fig. 1).

**Fig. 1:**
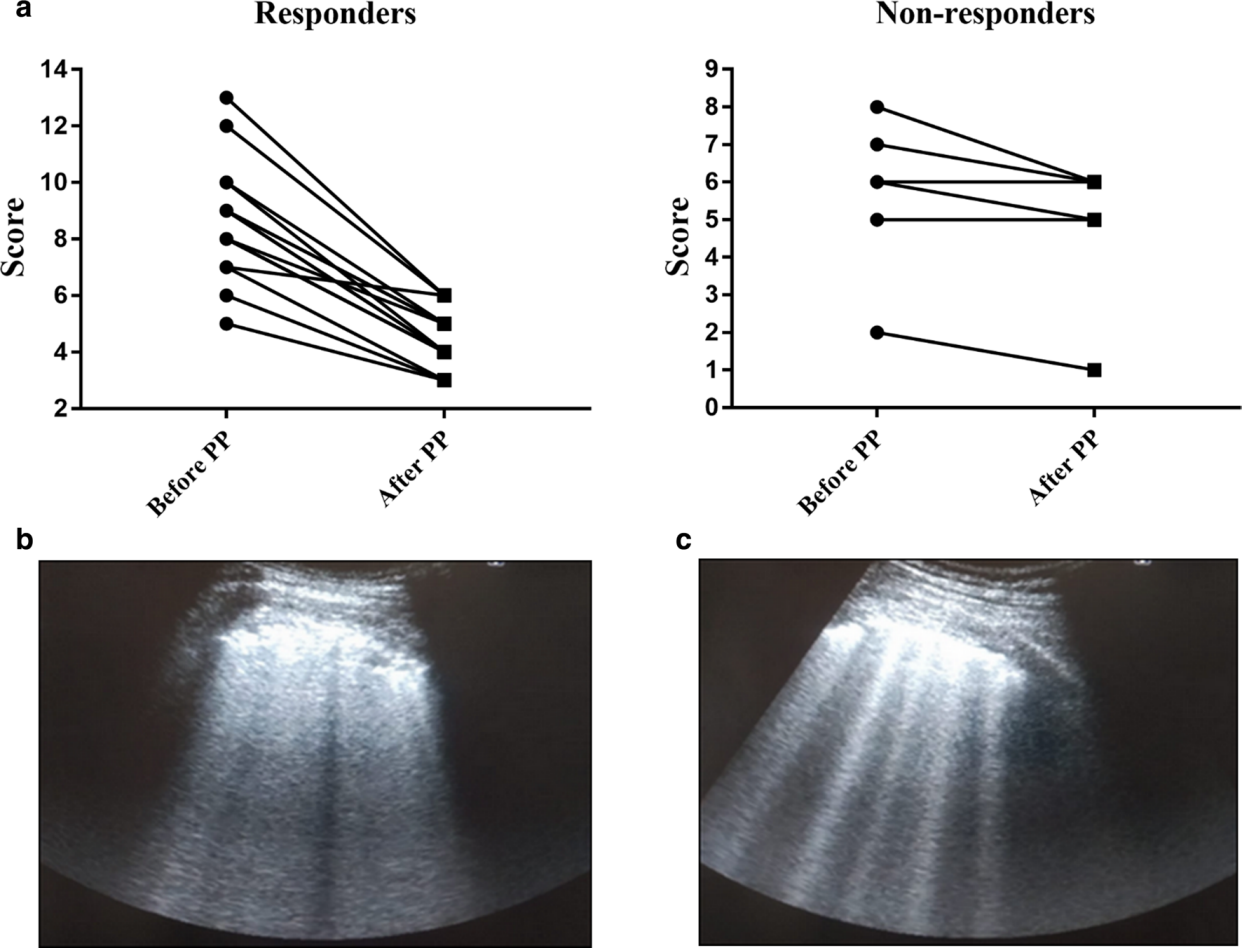
**a** Lung ultrasound scores of the posterior regions before and after prone positioning (PP) in responders (*n* = 16) and non-responders (*n* = 6). **b** Before prone positioning: irregular and broken pleural lines with multiple confluent B-lines. **c** After prone positioning: irregular and thickened pleural lines with several B-lines, predominate separated B-lines

Previous studies examined the changes of aeration by LUS in PP in intubated patients with ARDS not-associated with COVID-19 [5, 6]. Haddam et al. found that oxygenation response to PP was not correlated with a specific LUS pattern regardless of the focal or non-focal nature of ARDS [5]. However, Wang et al. demonstrated that aeration score changes assessed by LUS were significantly higher in the PP responder and survivor groups [6]. Our study demonstrated in awake non-intubated patients with COVID-19-associated ARDS the relationship between the pattern of lung changes (presence of areas with subpleural consolidations), their localization (posterior segments) as shown by LUS, and the response to PP.

In conclusion, in patients with severe COVID-19, response to PP probably depends on the extent and localization of lung tissue changes. The aeration changes assessed by LUS may be useful in prediction of oxygenation response to PP in awake non-intubated patients with COVID‑19‑associated ARDS.

## Data Availability

Data and materials can be obtained from the corresponding author upon the reasonable request.

## Abbreviations

PP: Prone position
ARDS: Acute respiratory distress syndrome
PaO_2_: Partial pressure of oxygen
FiO_2_: Fraction of inspired oxygen
PaCO_2_: Arterial carbon dioxide tension
SpO_2_: Oxygen saturation
CPAP: Continuous positive airway pressure
RR: Respiratory rate
HR: Heart rate
LUS: Lung ultrasound
